# Investigation of Local Conduction Mechanisms in Ca and Ti-Doped BiFeO_3_ Using Scanning Probe Microscopy Approach

**DOI:** 10.3390/nano10050940

**Published:** 2020-05-14

**Authors:** Maxim S. Ivanov, Vladimir A. Khomchenko, Maxim V. Silibin, Dmitry V. Karpinsky, Carsten Blawert, Maria Serdechnova, José A. Paixão

**Affiliations:** 1 CFisUC, Department of Physics, University of Coimbra, P-3004-516 Coimbra, Portugal; uladzimir@uc.pt (V.A.K.); jap@fis.uc.pt (J.A.P.); 2 MIREA—Russian Technological University “RTU MIREA”, Moscow 119454, Russia; 3 National Research University of Electronic Technology “MIET”, Zelenograd, Moscow 124498, Russia; sil_m@mail.ru (M.V.S.); dmitry.karpinsky@gmail.com (D.V.K.); 4 Scientific-Practical Materials Research Centre of NAS of Belarus, 220072 Minsk, Belarus; 5 Institute for Bionic Technologies and Engineering, I.M. Sechenov First Moscow State Medical University, Moscow 119991, Russia; 6 Scientific-Manufacturing Complex Technological Centre, Zelenograd, Moscow 124498, Russia; 7 Scientific and Educational Center “Nanotechnology”, South Ural State University, Lenin av. 76, Chelyabinsk 454080, Russia; 8 Institute of Materials Research, Helmholtz-Zentrum Geesthacht, Max-Planck-Straße 1, 21502 Geesthacht, Germany; carsten.blawert@hzg.de (C.B.); serdechnova@mail.ru (M.S.)

**Keywords:** BiFeO_3_, scanning probe microscopy, grain boundaries, domain walls, n- and p-type conductivity

## Abstract

In this work we demonstrate the role of grain boundaries and domain walls in the local transport properties of n- and p-doped bismuth ferrites, including the influence of these singularities on the space charge imbalance of the energy band structure. This is mainly due to the charge accumulation at domain walls, which is recognized as the main mechanism responsible for the electrical conductivity in polar thin films and single crystals, while there is an obvious gap in the understanding of the precise mechanism of conductivity in ferroelectric ceramics. The conductivity of the Bi_0.95_Ca_0.05_Fe_1−x_Ti_x_O_3−δ_ (x = 0, 0.05, 0.1; δ = (0.05 − x)/2) samples was studied using a scanning probe microscopy approach at the nanoscale level as a function of bias voltage and chemical composition. The obtained results reveal a distinct correlation between electrical properties and the type of charged defects when the anion-deficient (x = 0) compound exhibits a three order of magnitude increase in conductivity as compared with the charge-balanced (x = 0.05) and cation-deficient (x = 0.1) samples, which is well described within the band diagram representation. The data provide an approach to control the transport properties of multiferroic bismuth ferrites through aliovalent chemical substitution.

## 1. Introduction

The study of electronic states and energy band diagrams in relation to the crystal structure, ferroelectric, and magnetic properties remains one of the crucial tasks of condensed matter physics considering the interactions between the lattice, charge, and spin degrees of freedom [1,2,3,4,5]. There is a limited number of materials allowing such correlations to be simultaneously revealed. A model example is perovskite-type (ABO_3_) multiferroics exhibiting a coupling between the ferroelectric, elastic, and magnetic order parameters [6,7]. Among the known multiferroic perovskites, bismuth ferrite, BiFeO_3_, (BFO) is distinguished by the exceptionally high transition temperatures and large electric polarization, making it one of the most popular objects of magnetoelectric research [6,7]. The functional properties of BFO can be strongly affected by chemical substitution. For instance, doping of the material with Ca^2+^ yields: (i) ferroelectric state with spontaneous magnetization (the parent compound is an antiferromagnet) [8]; (ii) enhanced electromechanical response [9]; (iii) prominent electrochromic effect [10]; and (iv) modulation of electric conductivity arising due to the naturally produced oxygen vacancies [11,12]. The latter are known to largely determine the ferroelectric domain structure and electrical properties of BFO-based multiferroics [2,3,13,14,15,16].

In the last decade, the reasons underlying the phenomenon of increased conductivity at interfaces and domain walls (DW) in thin films of BiFeO_3_ [14,16], Pb (Zr, Ti) O_3_ [17,18,19,20], LiNbO_3_ [21], and hexagonal rare-earth manganites [22] have attracted immense interest. These investigations demonstrate that both positively and negatively charged defects are attracted to the DW region due to polarization divergence appearing between neighboring domains [2,11,14,23] which is also confirmed to be the main reason of conductivity in ‘improper’ ferroelectrics [22]. Despite the fact that ferroelectric ceramics are widely used in technological applications nowadays, the conductance of the polycrystalline materials is not well studied yet. There are several important findings reported for BFO-family compounds. In particular, it was found that all possible types of DW (71°, 109°, and 180°) in BFO ceramics are conductive at room-temperature [14,16,24,25,26,27,28]. Depending on the defect structure, both p- and n-type conductivity associated with the DW were found [16,29,30]. The p-type conductivity is due to charge transfer between Fe^4+^ and Fe^3+^ [2,16,25,26,27,28,29,30,31,32,33,34], while the n-type conductivity is linked to the formation of oxygen vacancies [16,25,26,27,28,29,30,31,32,33,34]. Some alternative mechanisms of conductivity in polycrystalline BFO suggesting the reduction of local bandgap at the boundaries [13,26] were also considered. Although the local conduction at DW is proven to be related to the internal domain structure: polarization state and tilting angle, the understanding of local conductivity variation at grain boundaries (GB) remains challenging. This results in an intensive debate regarding the general scenario describing the local and macroscopic conductivity in the polar polycrystalline perovskites. 

The current work aims to disclose the mechanisms of conductivity in BFO-based multiferroics depending on the defect structure introduced by aliovalent chemical substitution. To do this, conventional and conductive atomic force microscopy (AFM/c-AFM) as well as piezoresponse force microscopy (PFM) measurements of the mechanically-polished Bi_0.95_Ca_0.05_Fe_1−x_Ti_x_O_3−δ_ ceramics have been performed.

## 2. Experimental

Polycrystalline samples of Bi_0.95_Ca_0.05_Fe_1−x_Ti_x_O_3−δ_ (x = 0, 0.05, 0.1; δ = (0.05 − x)/2) were prepared by a conventional solid-state reaction method using the high-purity reagents Bi_2_O_3_ (99.9%, Sigma-Aldrich, St. Louis, MO, U.S.), CaCO_3_ (99%, Sigma-Aldrich, St. Louis, MO, U.S), Fe_2_O_3_ (99%, Sigma-Aldrich, St. Louis, MO, U.S), and TiO_2_ (99%, Sigma-Aldrich, St. Louis, MO, U.S). The starting materials taken in the stoichiometric cation ratio were mixed and pressed into pellets. The synthesis was carried out in air at 900 °C for 20 h. Further details of these materials’ synthesis and structural study are described in [35].

A commercial scanning probe microscope NTEGRA Prima (NT-MDT Spectrum Instruments, Moscow, Russia) equipped with stiff NSG30 probes (resonance frequency f = 300 kHz, force constant N = 40 N/m, tip curvature radius ~ 35 nm, conductive coating—PtIr (25 nm), Cr adhesion layer (25 Å)) were used. The ferroelectric domain visualization was performed under an applied AC voltage with VAC = 5 V and f = 100 kHz, while the c-AFM measurements were done by applying a DC voltage of ±5 V (x = 0 and x = 0.05) and ±10 V (x = 0.1). Current-voltage dependences were obtained within the DC bias voltage range from −15 to +15 V. In order to avoid the corrupted data related to the AFM probe fatigue during the scans, fresh tips for every new set of the c-AFM/PFM measurements were used. Furthermore, numerous iterations of scanning have been performed for different areas to properly analyze the conductivity of the samples.

## 3. Results and Discussion

Our previous structural study of these Ca, Ti-doped samples confirmed that the materials are single-phase and possess a noncentrosymmetric rhombohedral structure (space group R3c) specific to the pure bismuth ferrite [35]. It has been found that the pattern of changes in the lattice parameters of the Bi_0.95_Ca_0.05_Fe_1−x_Ti_x_O_3−δ_ compounds can be interpreted as suggesting the doping-induced elimination of oxygen vacancies at x ≤ 0.05 and the formation of cation vacancies at x > 0.05 [35]. It has also been shown that the readjustment of the defect structure associated with the mechanism of charge compensation in the aliovalent-doped BFO is accompanied by correlated changes in the morphology, ferroelectric/ferroelastic domain structure, and magnetic properties [35,36].

**Figure 1:**
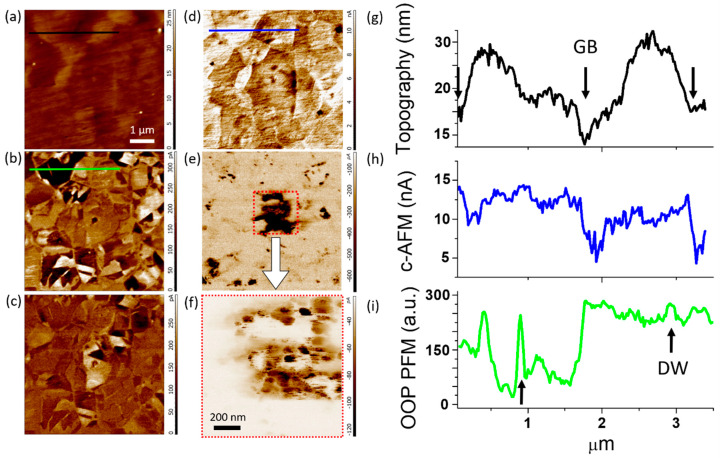
Scanning probe microscopy (SPM) measurements of the Bi_0.95_Ca_0.05_FeO_2.975_ sample: (**a**) surface topography, (**b**) out-of-plane (OOP) PFM response, (**c**) in-plane (IP) PFM response, (**d**) c-AFM response at positive DC bias voltage of 5 V, (**e**) c-AFM response at negative DC bias voltage of −5 V, (**f**) magnified c-AFM image of the area marked by the red dotted square in (**e**). Cross-section profiles for (**g**) topography, (**h**) c-AFM, and (**i**) OOP PFM. The arrows indicate the position of grain boundaries (GB) and domain walls (DW) on the corresponding cross-section profiles.

Figure 1 shows the results of the combined c-AFM/PFM measurements obtained for the Bi_0.95_Ca_0.05_FeO_2.975_ ceramics whose crystal structure and multiferroic properties have been described in Ref. [35]. The morphology of the sample is described as a conglomeration of micrometer-sized crystal grains (Figure 1a) demonstrating periodic changes in PFM signals which form complex patterns of ferroelectric domains (Figure 1b,c). The current detected during the c-AFM scanning reaches relatively large values of ~10 nA at the positive bias of 5 V (Figure 1d), thus suggesting that the main carrier type underlying the observed transport behavior must be electronic rather than ionic in nature [11]. The conductivity is of n-type. The scanning at negative bias voltage gives a very low signal with some local enhancements (Figure 1e,f) corresponding to local signal decays on the positive bias scan (Figure 1d). This might be indicative of the inhomogeneous distribution of chemical composition and related positively-charged defects. The dominant n-type conduction should originate from electron hopping between oxygen-vacancy-related defects [12]. The c-AFM signal distribution indicates an almost equipotential signal inside the grains with distinct changes only at GBs (Figure 1d). The conduction measurements performed within one grain reveal no difference between the domains and DWs (Figure 1g,i). In contrast, the grain boundaries conduction can be apparently distinguished via the cross-section profile lines overlaying the C-AFM and topography scan images (Figure 1g,h). The latter is consistent with the observation of the n-type conductivity in pure BFO, where the conduction is supported by thermally activated electrons from defect (oxygen vacancies) states [25,26,27,28,29,30,31,32,33,34]. It is worth noting that the conductivity of Ca/Ti-doped BFO can change from n- to p-type depending on defect structure and synthesis conditions [37].

**Figure 2:**
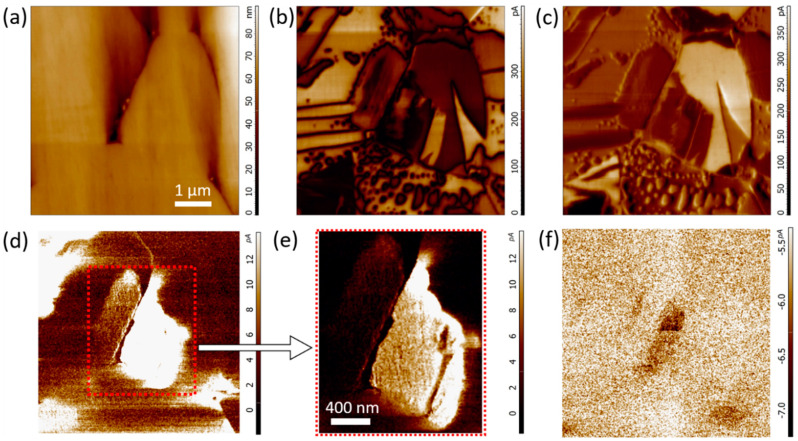
SPM measurements of the Bi_0.95_Ca_0.05_Fe_0.95_Ti_0.05_O_3_ sample: (**a**) surface topography, (**b**) OOP PFM response, (**c**) IP PFM response, (**d**) c-AFM response at positive DC bias voltage of 5 V, (**e**) magnified c-AFM image of the area marked by the red dotted square in (**d**), (**f**) c-AFM response at negative DC bias voltage of −5 V.

In the Bi_0.95_Ca_0.05_Fe_0.95_Ti_0.05_O_3_ compound, a deficit of positive charge introduced by the aliovalent replacement of Bi^3+^ with Ca^2+^ is expected to be fully compensated by Ti^4+^ ions and the oxygen content corresponds to the stoichiometric one. The charge balance-provided substitution favours the crystal grain growth [35,38], so the typical grain size increases up to 5–10 µm (Figure 2a). Consistent with the suggestion that the reduction of substitution-related lattice defects should increase the electrical resistivity, the material demonstrates at least a three order of magnitude lower conductivity (a few pA both at 5 V and −5 V) as compared with the Bi_0.95_Ca_0.05_FeO_2.975_ compound (Figure 2d–f). Scanning at the positive bias voltage reveals local areas (corresponding to grain boundaries, domain walls and some domains within a grain) (Figure 2a–c) with a slightly enhanced signal (~10 pA at 5 V) (Figure 2d,e) characteristic of oxygen vacancies-assistant conductivity observed at the DW of pure BFO [25,26].

**Figure 3:**
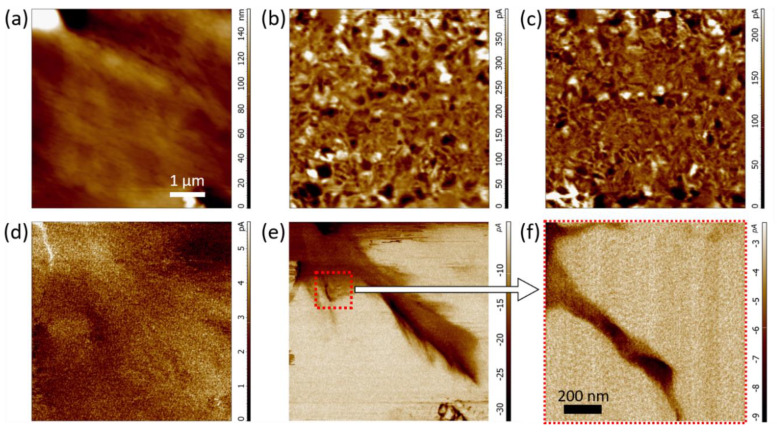
SPM measurements of the Bi_0.95_Ca_0.05_Fe_0.9_Ti_0.1_O_3.025_ sample: (**a**) surface topography, (**b**) OOP PFM response, (**c**) IP PFM response, (**d**) c-AFM response at positive DC bias voltage of 10 V, (**e**) c-AFM response at negative DC bias voltage of −10 V, (**f**) magnified c-AFM image of the area marked by the red dotted square in (**e**).

Above the threshold concentration (x = 0.05) corresponding to the compound with fully occupied oxygen sites (δ = 0), charge compensation is achieved through the creation of cation vacancies (which are proven to form extended lattice defects) [39,40]. The over-compensatory Ti^4+^ doping results in a dramatic decrease of both the average grain size and typical ferroelectric domain width (Figure 3a–c) as previously observed [35]. The c-AFM scanning reveals very weak response at positive bias voltage of up to 50 V (Figure 3d), while the application of negative bias can result in the local signal enhancement (about −20 pA at −10 V) randomly appearing over the sample surface (Figure 3e,f). The regions with increased p-type conductivity should be attributed to grains enriched by Ti doping-induced lattice defects. The related defect structure suggests the formation of highly-strained nanorod precipitates and antiphase boundaries exhibiting a drastic deviation from the average crystal structure and chemical composition [39,40]. Such nanoscale defects’ segregation [41] seems to prevent the formation of conductive pathways and conductivity of the Bi_0.95_Ca_0.05_Fe_0.9_Ti_0.1_O_3.025_ compound remains as low as that of Bi_0.95_Ca_0.05_Fe_0.95_Ti_0.05_O_3_.

**Figure 4:**
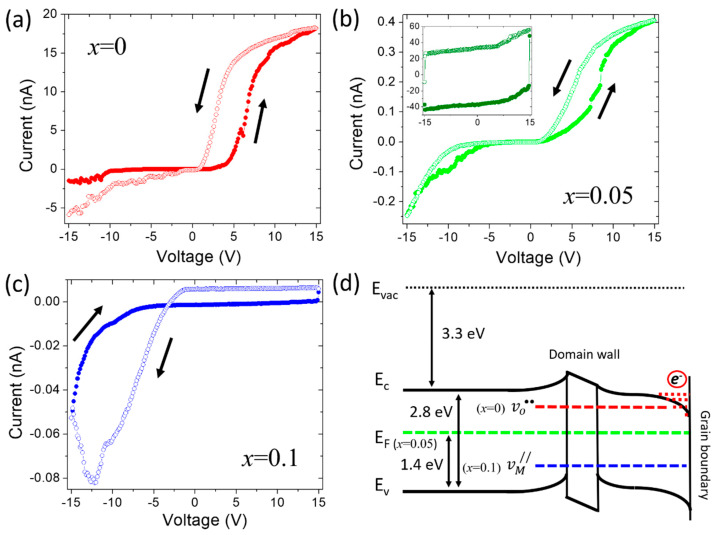
Local current-voltage dependencies for the Bi_0.95_Ca_0.05_Fe_1−x_Ti_x_O_3−δ_ samples, as measured within conductive areas: (**a**) x = 0, (**b**) x = 0.05, (**c**) x = 0.1. The inset shows the dependence characteristic of insulating regions (the vertical scale is in pA). (**d**) The band diagram representation considering the influence of grain boundaries and domain walls on the energy band bending depending on the chemical composition. The values representing the Fermi level (1.4 eV), bandgap (2.8 eV), and the electron affinity (3.3 eV) of BFO were taken from Refs. [13,15,42].

In order to demonstrate the proper conductivity of each sample, the local current-voltage curves were measured inside the merged grain/domain area possessing maximum c-AFM signal (Figure 4a–c). The obtained I-V dependences demonstrate the rectifying diode behavior, as was previously reported for pure and doped BFO [3,16,26]. The data obtained for the sample with *x* = 0 indicate that the forward diode direction coincides with that of the electrical polarization, thus confirming that carriers are n-type (Figure 4a). The anion/cation balanced sample (x = 0.05) shows the forward and backward diode current-voltage characteristic specific to p-n junction (Figure 4b). The oxygen-enriched sample (x = 0.1) exhibits the backward diode behavior indicating that carriers are of p-type. It also demonstrates a drastic increase of the reverse current which can be associated with electrostatic forces caused by Schottky emission from the tip (Figure 4c).

The c-AFM and I-V measurements suggest that the aliovalent substitution in the Bi_0.95_Ca_0.05_Fe_1−x_Ti_x_O_3−δ_ series gives rise to the mechanisms of conductivity being largely similar to those of donor/acceptor-doped semiconductors. To illustrate that, the band diagram approach can be proposed. The chemical substitution affects the energy band diagram adding the donor level ($v_{o}^{••}$) at x = 0 and acceptor level ($v_{M}^{//}$) at x = 0.1, while the Fermi energy (E_F_) of the charge-balanced sample (x = 0.05) maintains at equilibrium state (Figure 4d). It is important to mention that the local changes in the energy offset at DW and GB represent different influence on energy states inducing the band bending. This is mainly due to the difference in space charge distribution, namely a local imbalance in charge neutrality between electrons/electron holes and ionized defects [11,19,42]. GB can be presented as a junction determining the perovskite’s work function with respect to the vacuum electrostatic potential (Figure 4d). DW joins the areas with different polarizations (similar to p-n-p or n-p-n junctions in a semiconductor) in which the bands vary continuously since the polarization gradient associated with DW is spatially distributed and only perturbs the system changing the Schottky barrier due to the defect surface states (Figure 4d). This approach describes well the increased conductivity along the GB in the x = 0 sample (Figure 1d), where the effect of the inverted population of mobile charge carriers is expected (the additional electrons at the grain boundary drawn in Figure 4d).

## 4. Conclusions

To sum up, the study of conductivity in the Bi_0.95_Ca_0.05_Fe_1−x_Ti_x_O_3−δ_ (x = 0, 0.05, 0.1) ceramics as a function of the defect and ferroelectric domain structure has been performed. The obtained results demonstrate how the chemical modification changes the local transport behavior associated with grain boundaries, domains, and domain walls depending on the type of mobile defects. The band diagram approach properly supports the experimental data, confirming that the conductivity of the doped BFO can be mediated by charge carriers induced by the chemical substitution. The results shed light on the mechanism of the substitution-driven conductivity in polycrystalline BFO, which is particularly important for the development of nanoelectronic devices based on multiferroic ceramic materials.
